# The Efficacy of Little Lovely Dentist, Dental Song, and Tell-Show-Do Techniques in Alleviating Dental Anxiety in Paediatric Patients: A Clinical Trial

**DOI:** 10.1155/2021/1119710

**Published:** 2021-05-23

**Authors:** Hira Abbasi, Muhammad Saqib, Rizwan Jouhar, Abhishek Lal, Naseer Ahmed, Muhammad Adeel Ahmed, Mohammad Khursheed Alam

**Affiliations:** ^1^Department of Operative Dentistry, Altamash Institute of Dental Medicine, Karachi 75500, Pakistan; ^2^Department of Restorative Dentistry and Endodontics, College of Dentistry, King Faisal University, Al-Ahsa 31982, Saudi Arabia; ^3^Prosthodontics Unit, School of Dental Sciences, Health Campus, Universiti Sains Malaysia, 16150 Kubang Kerian, Kota Bharu, Kelantan, Malaysia; ^4^Department of Prosthodontics, Altamash Institute of Dental Medicine, Karachi 75500, Pakistan; ^5^Department of Preventive Dentistry, College of Dentistry, Jouf University, Sakaka, Al Jouf 72345, Saudi Arabia

## Abstract

**Introduction:**

Dental anxiety is a common occurrence in patients undergoing dental treatments, especially in children. The success in paedriatric dental treatments and patient comfort depends on controlling the level of patient's anxiety in clinical settings. This study is aimed at evaluating the efficacy of different techniques applied for the reduction of dental anxiety in paediatric patients. *Material and Methods*. One hundred and sixty participants were divided into 4 groups; each group having 40 patients as follows: group I: mobile application “little lovely dentist,” group II: YouTube® “dental video songs,” group III “tell-show-do,” and group IV “control.” Dental prophylaxis treatments were provided to all the participants. Initial anxiety levels were noted during the patient's education phase by measuring heart rate with pulse oximeter and distress level with facial image scale, at the same time in each group, respectively. The postoperative anxiety was noted later with the same methods, after the application of anxiety reduction techniques. The data obtained were entered in the statistical package for the social sciences software, version 25. One-way ANOVA and paired *t*-test for matched groups were used to compare mean values of the 4 groups, in this study to determine their effectiveness. A *p* value of ≤0.05 was considered as statistically significant.

**Results:**

The mean age of patients in group 1 was 6.8 ± 2.1 years, group 2: 8.15 ± 2.27 years, group 3: 7.5 ± 2.3 years, and group 4: 7.27 ± 1.68 years. The intragroup comparisons of heart rate and facial image scores have shown a significant difference in before and after dental treatment procedures. Marked reduction in heart rate and facial image scale scores were found in patients belonging to group 1 (mobile applications) and group 2 (dental video songs). An increase in heart rate and facial image scale scores was seen in group 3 (tell-show-do) and the control group.

**Conclusion:**

The paediatric dental anxiety is a common finding in dental clinics. Behavior modification techniques like smartphone applications, “little lovely dentist,” and “dental songs” can alleviate dental anxiety experienced by paediatric patients. The “tell-show-do” technique although most commonly used did not prove to be beneficial in the reduction of the anxiety levels.

## 1. Introduction

One of the crucial determining factors associated with desired outcomes after dental treatment in paediatric patients is dental anxiety control. Fear and anxiety are particularly high among paediatric patients visiting the dentists, and its proportion is substantial which often hinders optimal dental care for the children [1]. Generally, anxiety is explained as a fearful reaction to various stimuli such as dental treatment and it is often categorized as feeling nausea, vomiting, increased blood pressure, high heart rate, and palpitations [2].

The children who suffer from dental anxiety and fear range from 5 to 33% throughout the world and are ranked as the fourth common fear [2, 3]. The first experience that the child face during his initial visit to the dentist usually determines his future willingness for dental treatment [4]. Therefore, a pleasant experience normally generates a sense of ease and trust in the dentist, while a traumatic experience leads to avoidance of visiting the dentist if required again, hence shows the importance of past dental experience [5].

Previously, many researchers have concluded that between 50 and 80% of adults have some degree of dental anxiety that ranges from mild to severe [6]. Moreover, more than 20% of the patients did not visit their dentist regularly and from 9 to 15% of anxious patients avoid any sort of dental treatment provided to them [6]. The perception with which the patient visits the dentist has a substantial effect on the levels of anticipated anxiety, for example, if the patient already comes with the anticipation of predetermined anxiety, then the treatment will indeed turn out to be unpleasant and anxious for the patient [7, 8].

Children who suffer from dental anxiety avoid any sort of dental treatment provided to them, which leads to poor oral hygiene, and if unaddressed, problems like missing teeth and decayed teeth develop further in life, and ultimately leading to problems making treatment planning difficult [9, 10]. The child follows the footprints of their parents, if the parents had a traumatic experience during a dental visit and they shared it with the child, it creates a negative image of the dentist in the child's mind. Hence, during the first visit, it may create an unfavorable environment for successful dental treatment of the child [11, 12].

Due to the substantial effect of dental anxiety in paediatric patients, many methods have been devised to alleviate or eliminate such anxieties of the patients. Owing to the fondness of the children with mobile applications and games, a dental app has been created named “little lovely dentist” which primarily engages the little ones on how the dentist will perform different treatments on them such as scaling, fillings, extractions, and much more [13]. Secondly, children are also occupied with watching cartoons particularly, which led to the development of various informative animated cartoon videos explaining the dental treatment of the children without showing any invasive treatment that might trigger fear and anxiety. Thirdly, and most commonly used technique in paediatric patients is the tell-show-do method. This method consists of verbally explaining the treatment to the patient, then showing the use of different dental instruments, and finally performing the procedure on the child [14].

Sitting on the dental chair is itself a state of fear for any patient regardless of age, and this anxiety is particularly high in children. So, the dentists employ various techniques along with the above-mentioned such as behavioral management and psychological methods to counteract anxiety [15].

This study is aimed at determining the efficacy of 3 techniques, “little lovely dentist,” “dental video songs,” and “tell-show-do” in reducing dental anxiety, by measuring heart rate and facial image scale (FIS) scores, before and after the dental procedures.

## 2. Materials and Methods

### 2.1. Study Setting and Sample Size

This study was carried out from August 2020 till December 2020 in the department of paediatric dentistry at Altamash Institute of Dental Medicine, Pakistan. OpenEpi® software was used to calculate the sample size by keeping a 95% confidence interval and the desired percentile of 50% [16]. The estimated sample size was 40 patients per group.

### 2.2. Ethical Consideration and Participant Recruitment

The ethical and review board of Altamash Institute of Dental Medicine (AIDM/EC/07/2020/06) approved this study. The trial is registered under “clinicaltrials.gov” (United States National Library of Medicine) with identifier number (NCT04833478). The voluntary participation and refusal at any point during the trial were sought out. Informed and written consent was taken from the patient's parents or guardians before including them in the trial. The inclusion criteria were children of age 6 to 11 years who showed a willingness to take part in the study with no previous dental treatment or visit history and whose behavior could be rated as positive (+) or negative (-) based on “Wright's modification of the Frankl behavior rating scale” [17]. Medically compromised children, those with disabilities, severe pain, facial swelling, and trauma were excluded from this study. Overall, 210 paediatric patients were assessed for this study. Fifty patients were excluded due to either failure to fulfill inclusion criteria or nonwillingness to take part in the study.

### 2.3. Grouping and Randomization of Participants

**Figure 1:**
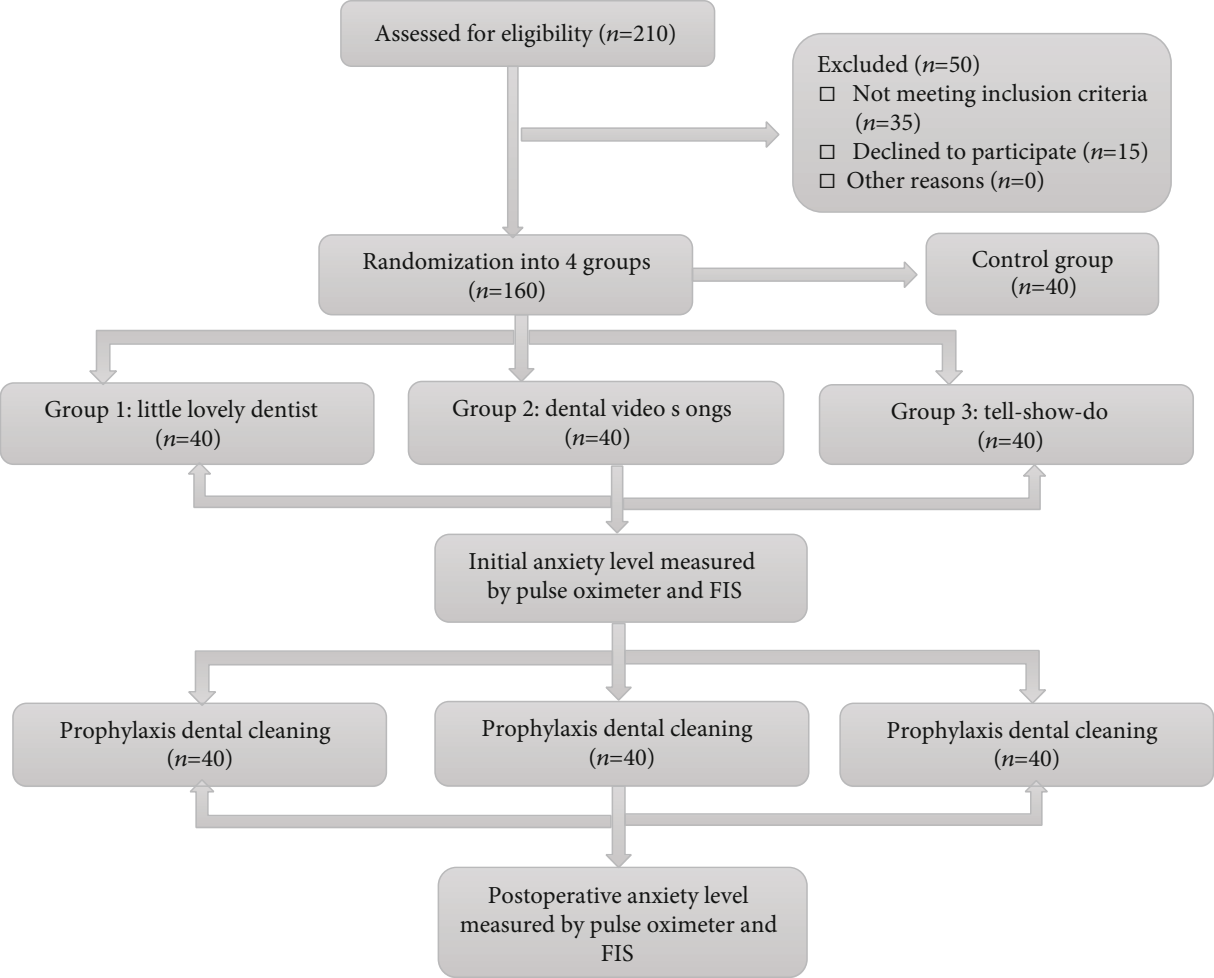
CONSORT flow diagram of the study.

**Figure 2:**
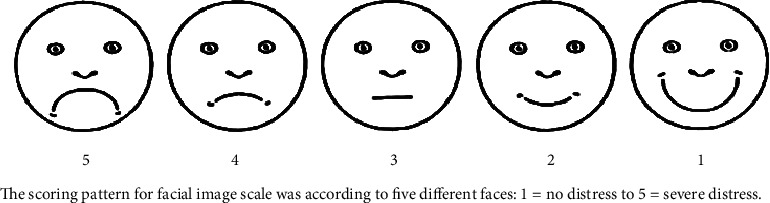
Facial image scale (FIS). The scoring pattern for facial image scale was according to five different faces: 1 = no distress to 5 = severe distress.

After demographic data collection and screening of the child, 160 participants were divided into 4 groups by randomization through the lottery method, with each group having 40 patients as follows: group I: mobile application “the little lovely dentist” technique, group II: “dental video songs” technique, group III: “tell-show-do” technique, and group 4: “control” (Figure 1). The initial anxiety levels were noted before the start of treatment procedures during the education stage in all patients. The heart rate was measured with a pulse oximeter (Finger Pulse Oximeter YP-1, respectively) and simultaneously distress level by using facial image scale (FIS) [18]. In FIS, score 1 denotes “no distress” and 5 denotes “severe distress” (Figure 2). Dental prophylaxis (cleaning) treatments were provided to all the participants as explained during education. The heart rate and FIS scores were again recorded from the respective study groups immediately after the treatment was provided.

### 2.4. Application of Anxiety Reduction Protocols

The application “little lovely dentist” has been developed by Leaf cottage software and Shanghai Edaysoft Co., Ltd., which is available on App Store and Google Play software to download it from, respectively. The application consists of various activities which include restorations, fissure sealants, extractions, brushing, and playfully explaining oral hygiene methods to the child. For dental song, various songs are available on YouTube for children which joyfully explains to the children about different dental treatments that are available, along with oral hygiene maintenance methods and their importance with it. For the tell-show-do (TSD) technique, in the “tell” part, there is a verbal explanation for the dental procedure appropriate to the developmental level of the child. In the “show” part, demonstration of various instruments was given to the child in a friendly manner to build their confidence, and lastly, in the “do” part, the dentist performed the desired procedure exactly as explained to the child. Lastly, in the control group, no behavior modification technique was used; heart rate and facial image scale scores were measured in the same way as for the above-mentioned groups.

### 2.5. Statistical Analysis

For the data analysis, the statistical package for the social sciences software (IBM, SPSS Statistics, version 25, Chicago, Illinois, United States) was used. In each group, before and after recording, the heart rate and FIS were compared to check the effectiveness of that particular technique in alleviating or eliminating the child's dental anxiety. Descriptive statistics along with a one-way ANOVA test and a paired *t*-test for matched groups were used to compare the mean values of the 4 groups in this study to determine their effectiveness. A *p* value of ≤0.05 was considered as statistically significant.

## 3. Results

The distribution of males and females in group 1 was 21 males and 19 females, group 2: 19 males and 21 females, group 3: 17 males and 23 females, and group 4: 22 males and 18 females. The mean age of participants in group 1 was 6.8 ± 2.1, group 2: 8.15 ± 2.27, group 3: 7.5 ± 2.3, and group 4: 7.27 ± 1.68, as presented in Table 1.

The mean heart rate of participants with little dentist technique was 106.4 ± 7.5, dental song was 105.3 ± 6.9, tell-show-do was 104.8 ± 11.6, and control was 102.9 ± 5.3. Among the four groups, group 1 showed maximum reduction in anxiety levels, then group 2 but groups 3 and 4 showed an increase in the anxiety levels. According to the age, higher anxiety levels were found in the young age group as compared to the older age group (*p* = 0.001). Additionally, a statistically significant difference was found in before and after heart rate in group 1 (*p* = 0.002), group 2 (*p* = 0.001), and group 4 (*p* = 0.013) while no difference was seen in group 3 (*p* = 0.677), respectively, as mentioned in Table 2.

**Figure 3:**
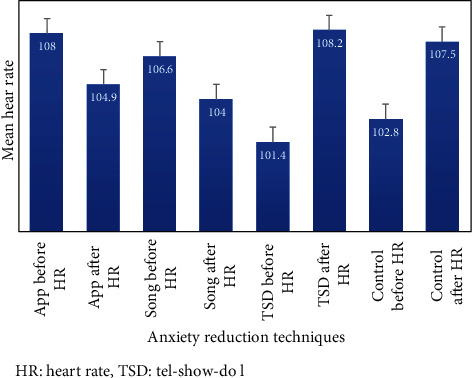
Distribution of mean heart rates of the patients before and after procedures using different techniques (*n* = 120). HR: heart rate; TSD: tell-show-do.

Furthermore, among the different groups, the little lovely dentist application was found to the most effective in decreasing the level of anxiety as denoted by the heart rate in the paediatric patients, with dental song also showing a similar decrease in levels of anxiety. However, the tell-show-do technique is the most frightful technique for children while explaining and showing different dental techniques as shown in Figure 3.

For the intragroup comparison of subjective anxiety, the comparison of facial image scale score (FIS) with anxiety reduction techniques is described in Table 3. The mean FIS score in participants with little dentist technique was 2.66 ± 0.97, dental song was 2.63 ± 0.90, tell-how-do was 2.95 ± 0.85, and control group was 3.21 ± 1.08. Moreover, a statistically significant difference was found in before and after FIS score in group 1 (*p* = 0.032), group 2 (*p* = 0.036), group 3 (*p* = 0.001), and group 4 (*p* = 0.013), respectively. Regarding the facial image scores, participants belonging to group 1 (little lovely dentist) and group 2 (dental song) demonstrated a reduction in the level of anxiety by selecting a lower score after treatment as compared to the scores selected before the treatment. In contrast to this, group 3 (tell-show-do) patients reported higher anxiety levels by selecting a higher anxiety score after the dental procedure as compared to them before treatment scores.

## 4. Discussion

Dental anxiety has categorically been one of the crucial sources of problems for many patients as well as the dentists as it leads to many problems including unnecessary delays in the treatment, along with less-than-optimal treatment for the patients as there is a lack of compliance which at times frustrates the dental surgeon.

Age is a critical factor regarding compliance with the dental procedures along with dental anxiety experienced. In our study, it was found that dental anxiety was most profoundly present in children of lower age groups as compared to those in higher age groups. These results correlate with previous studies carried which report the prevalence of higher dental anxiety in younger age groups [19, 20], although this is not always applicable as elders' individuals also tend to experience dental anxiety as well primarily due to their past traumatic experiences with the dentists [21].

The very first treatment that the child undergoes with the dentist is of vital importance as it is the determining factor for further dental treatments later on in life [22]. Literature states that children who visit the dentist for the very first time often exhibit poor behavior and compliance, which corresponds to the findings in this study [23]. If the initial dental treatment is not pleasant for the child, this will create a lack of trust with any dentist for any dental treatment further on in life, compromising the oral health of such individuals [24].

Gaining a child's trust regarding any dental procedure often leads to the successful completion of most dental treatment along with a positive response for further treatments as well. A child-friendly environment such as cartoon-shaped masks, scrubs, and instruments of the dentists often relieves much of the anxiety and stress faced by the newly visiting paediatric patients [25]. Making the ambiance of the dental clinic is a vital factor for smooth handling of the paediatric dental needs.

Visual representation of the treatment in the form of dental applications that is to be performed is the most effective method to reduce paediatric anxiety, as shown in our study. Similar to this was observed with patients shown the dental song technique along, but the tell-show-do technique further exacerbated anxiety in the already anxious child with further increase in their heart rates after performing it, respectively. This corresponds to the previous studies which also fail to demonstrate any significant reduction in anxiety levels of the paediatric patients after demonstrating the “tell-show-do” technique [26, 27].

In today's world, most households have different electronic devices such as smartphones, tablets, and televisions through which children get introduced to various aspects of life which include medical treatment cartoons, more specifically dental treatments. The use of such electronic devices by children is also on the rise [28]. Cartoon songs have been a source of great enjoyment for almost all children, using the dental song to reduce dental anxiety of the patients responded positively as the anxiety levels decreased as shown in our study. Currently, different applications have been developed or are being currently developed for use in medical and dental fields to educate the patients regarding the procedures in the hope of decreasing anxiety of the patients [29].

All of the paediatric patients that presented to the outpatient department for their dental treatment exhibited varying levels of anxiety related to dental treatment mainly due to it being the first time being exposed to such an environment along with experiences shared by their relatives and friends who might not have had the best of dental experiences, which further worsens the already anxious patients [30, 31].

Patients in group 1 (the little lovely dentist) showed a marked reduction in their anxiety levels as compared to group 2 (dental song) and group 3 (tell-show-do). The application developed includes various interactive and joyful activities for the patient to perform on the electronic devices which produce a similar environment and sounds of different dental procedures which the child will ultimately go through.

Regarding the assessment of dental anxiety before and after treatment using facial image scale (FIS), facial image scale scores decreased for group 1 and group 2 but group 1 (little lovely dentist) outperformed group 2 in producing the most significant decreased FIS scores. However, on the other hand, the tell-show-do technique produced the most significant increase in the facial image scale scores of the paediatric patients as compared to the other groups.

According to the findings of this study, the dental application is found to be the most effective method of reducing dental anxiety in patients. These results correspond to a similar study being carried out by Patil et al. [13] and Shah et al. [32]; they demonstrate similar findings when compared with the “tell-show-do” technique.

Despite the strengths of this study which include a good sample size of each group and multiple variables used to assess paediatric dental anxiety, the present study has some limitations. The unequal gender distribution in each group can be one of the two possible limitations of this study, the other being a smaller range of age group selected. Therefore, further clinical trials with higher age groups and comparison of the behavior modification techniques applied in this study are recommended with other contemporary methods.

A better understanding and use of different behavior modification techniques by the dentists will aid in reducing or eliminating dental anxiety not only of paediatric patients but also of adults, as optimum treatment depends on a well-controlled and stress-free environment for both the patient and the dentist.

## 5. Conclusions

The paediatric dental anxiety is a common finding in dental clinics. Behavior modification techniques like smartphone applications, “little lovely dentist,” and “dental songs” can alleviate dental anxiety experienced by paediatric patients. The “tell-show-do” technique although most commonly used did not prove to be beneficial in the reduction of the anxiety levels.

## Figures and Tables

**Table 1:** Demographic characteristics of the patients of different groups.

Groups	Variables	Mean and standard deviation
Little lovely dentist	Age	6.8 ± 2.1
Dental song	Age	8.15 ± 2.27
Tell-show-do	Age	7.5 ± 2.3
Control	Age	7.27 ± 1.68

**Table 2:** Comparison of heart rate and level of anxiety among the study groups (*n* = 120).

Anxiety reduction techniques		*N*	Mean	Standard deviation	*p* value
Little lovely dentist	Before heart rate	40	107.9	8.2	0.002
	After heart rate	40	104.9	6.8	
Dental song	Before heart rate	40	106.6	6.1	0.001
	After heart rate	40	104	7.6	
Tell-show-do	Before heart rate	40	101.4	15.6	0.677
	After heart rate	40	108.2	7.5	
Control	Before heart rate	40	102.8	5.3	0.013
	After heart rate	40	107.5	5.9	

**Table 3:** The comparison of FIS scores with anxiety reduction techniques in participants (*n* = 120).

Anxiety reduction techniques		Mean	*N*	Standard deviation	*p* value
Little lovely dentist	Application FIS before	2.80	40	1.06	0.032
	Application FIS after	2.52	40	0.87	
Dental song	Song FIS before	2.80	40	0.96	0.036
	Song FIS after	2.47	40	0.84	
Tell-show-do	Tell-show-do FIS before	2.60	40	0.74	0.001
	Tell-show-do FIS after	3.30	40	0.96	
Control	Control FIS before	2.91	40	0.92	0.013
	Control FIS after	3.52	40	1.24	

FIS: facial image scale; *N*: number of participants.

## Data Availability

The raw data used to support the findings of this study are included within the article.
